# The role of oxytocin on self‐serving lying

**DOI:** 10.1002/brb3.1518

**Published:** 2020-01-13

**Authors:** Cornelia Sindermann, Ruixue Luo, Benjamin Becker, Keith M. Kendrick, Christian Montag

**Affiliations:** ^1^ Department of Molecular Psychology Institute of Psychology and Education Ulm University Ulm Germany; ^2^ The Clinical Hospital of Chengdu Brain Science Institute MOE Key Laboratory for Neuroinformation University of Electronic Science and Technology of China Chengdu China

**Keywords:** lying, OXTR, oxytocin, self‐serving lying

## Abstract

**Introduction:**

The effects of intranasal administration of the neuropeptide oxytocin on social cognition and behavior are highly specific. Potentially situational and personal variables influence these effects. The aim of the present study was to investigate effects of oxytocin administration on self‐serving lying, including situational effects.

**Methods:**

A total of 161 adult males participated in a randomized double‐blind placebo‐controlled between‐subject intranasal oxytocin administration (24 international units) study. Self‐serving lying was assessed using three subsequent rounds of the die‐in‐a‐cup paradigm, in which different degrees of lying can be implemented by the participants that can be determined on group level.

**Results:**

Oxytocin administration seemed to promote self‐serving lying, particularly in the third (last) round and only to a certain degree (not to the maximum possible).

**Conclusions:**

Our findings demonstrate that oxytocin administration can promote self‐serving lying when given repeated opportunities to lie. Moreover, exploratory results presented in the Supplementary Material indicate that the sensitivity to the effects of intranasal oxytocin in this domain might be moderated by individual differences in the oxytocin receptor gene.

## Graphical abstract

The effects of intranasal administration of the neuropeptide oxytocin on social cognition and behavior are highly specific. Our findings demonstrate that oxytocin versus placebo administration can promote self‐serving lying to a certain degree when given repeated opportunities to lie.

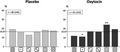

## 1 INTRODUCTION

While honesty is an important moral behavior (see, e.g., RAL‐Institut (2011), as cited in Statista (2011a, 2011b)) and many people claim for themselves to be honest (e.g., Mazar, Amir, & Ariely, 2008), lying is still prevalent and often generates public indignation and criticism. Results of a diary study indicate that people lie in around 20%–30% of their everyday social interactions (DePaulo, Kashy, Kirkendol, Wyer, & Epstein, 1996). In this context, it is important to note that different kinds of lies exist and that reasons to lie are manifold (DePaulo et al., 1996); hence, not all lies are considered as immoral or socially unacceptable (Chrismon.de (2008), as cited in Statista (2008)). But lying to enhance one's own payoffs or reduce one's own costs, namely self‐serving lying, is particularly seen as immoral and a violation of social norms since it can disrupt social relations by damaging interpersonal trust or results in a cost for others.

Despite the frequent occurrence of (self‐serving) lying in everyday life, surprisingly little is known about its biological underpinnings. An interesting candidate for further investigation of the biological underpinnings of self‐serving lying might be the hypothalamic neuropeptide oxytocin (OXT). Two previous studies have reported that intranasal administration of OXT increases lying for financial gain when it benefits an in‐group (including oneself; Shalvi & De Dreu, 2014), or in a competitive situation (in contrast to a noncompetitive setting) in association with conformity to perceived deceptiveness of others (Aydogan, Jobst, D'Ardenne, Müller, & Kocher, 2017). Additionally, self‐serving effects of intranasal OXT administration to enhance one's own payoff are also reported for other behaviors than lying (e.g., Xu et al., 2019). However, using a coin‐toss (prediction) task in which participants have only two possible choices—to “lie” or “be honest”—neither of the previously mentioned OXT administration studies on lying behavior found evidence for effects on pure self‐serving lying in the absence of a social benefit or justification (e.g., when lying did not serve the in‐group). Dishonesty is, however, subject to different gradations, and this can be investigated using the so‐called die‐in‐a‐cup paradigm where subjects have the opportunity to lie to varying degrees (Fischbacher & Föllmi‐Heusi, 2013; Gächter & Schulz, 2016). This paradigm has, for example, been used to demonstrate that testosterone administration can reduce self‐serving lying in males (in the absence of a social justification; Henderson, Thoelen, Nadler, Barraza, & Nave, 2018; Wibral, Dohmen, Klingmüller, Weber, & Falk, 2012), and previous evidence suggests that testosterone exhibits opposing effects on social cognitive functions as compared to OXT (Crespi, 2016).

Moreover, previous studies have not addressed the question of whether OXT effects might vary with an increasing number of opportunities to lie. Clearly, the decision to exhibit dishonesty can change when there is a repeated chance to lie, dependent upon the actual outcome in the previous rounds (low vs. high gain), the extent to which participants are convinced that lying would be (or would not be) detected and/or punished, as well as changes in the affective response toward lying (“getting used to it”). In line with the latter hypothesis, a previous experimental study reported that self‐serving lying (which was not detected/punished) increased with repeated opportunities to lie. Moreover, this increase in self‐serving lying over trials was associated with attenuated amygdala activation (Garrett, Lazzaro, Ariely, & Sharot, 2016). The authors hypothesized that the latter association might be explained by a reduction in the emotional response or the affective assessment and salience of self‐serving lying. Importantly, the amygdala is considered as key neural substrate that mediates the social cognitive and emotional effects of intranasal OXT (see also previous works described in Kendrick, Guastella, and Becker (2017)). As such, empirical studies show that OXT treatment among others reduces amygdala reactivity toward fearful faces (Spengler et al., 2017) and decreases amygdala activation while approach of angry faces (Radke et al., 2017). Moreover, the overarching social salience hypothesis of OXT suggests that—partly in interaction with dopamine—(exogenous administered) OXT influences social salience processes via effects on the amygdala (Shamay‐Tsoory & Abu‐Akel, 2016).

To this end, the present study aimed at investigating the effects of intranasal administration of OXT on self‐serving lying when participants are given the chance to lie repeatedly without negative consequences using repeated rounds of the die‐in‐a‐cup paradigm. Given (a) the self‐serving effects of intranasal OXT administration to enhance one's own payoff found for other behaviors than lying (Xu et al., 2019) and (b) the reduction of self‐serving lying found by testosterone administration (Henderson et al., 2018; Wibral et al., 2012), it is conceivable that OXT administration would enhance self‐serving lying. However, one also needs to take into account the nonsignificant findings on self‐serving lying (in the absence of a social justification) in previous OXT administration studies (Aydogan, Jobst, et al., 2017; Shalvi & De Dreu, 2014), even if there are marked differences between the tasks of these previous studies and the present one. Therefore, we aimed at investigating whether OXT would enhance self‐serving lying behavior in the die‐in‐a‐cup paradigm. Additionally, given the association between OXT and amygdala (de‐)activation as well as the association between amygdala (de‐)activation and escalation of lying, we aimed at investigating a potential effect of OXT on self‐serving lying when participants have repeated opportunities to lie undetectably.

Moreover, it needs to be mentioned that, originally, the study was also conducted to further investigate potential moderating effects of variations in the oxytocin receptor (OXTR) gene. However, given the rather low final sample size (and the special way of analyses conducted  to analyze the die‐in‐a‐cup paradigm), we decided to include the genetic background, analyses, results, and discussion in the Supplementary Material, only. A replication of these results in future works is necessary.

We are of the opinion that an investigation of underpinnings of self‐serving lying behavior is of great importance given the frequency of this behavior in daily life as well as the tremendous costs, which can be caused by extreme cases of such a behavior (e.g., the Ponzi scheme fraud of Bernard Madoff, which was estimated to cost around 65 billion dollars [https://www.reuters.com/article/us-madoff/madoff-mysteries-remain-as-he-nears-guilty-plea-idUSTRE52A5JK20090311?pageNumber=2%26virtualBrandChannel=0%26sp=true]). A focus on the OXT system is of specific importance given recent discussions on the therapeutic potential of intranasal OXT as novel treatment for disorders characterized by pronounced deficits in social behavior, including autism as well as borderline personality disorders (Kendrick et al., 2017). Particularly in the clinical context, it is of critical importance to evaluate whether intranasal OXT may also amplify self‐serving behavior rather than only enhancing prosocial behavior. Moreover, several context and person variables have been considered as factors that influence the effects of intranasal OXT on social behavior (Bartz, Zaki, Bolger, & Ochsner, 2011). The present work is an attempt to take into account some of these variables.

## 2 MATERIAL AND METHODS

### 2.1 Participants

Participants were first recruited for the Chengdu Gene Brain Behaviour Project (CGBBP) where they provided buccal cells for genotyping as well as completed a number of questionnaires including the HEXACO‐PI‐R Honesty‐Humility personality scale (Lee & Ashton, 2018). After participation in the CGBBP, male participants were invited to participate in a randomized double‐blind placebo‐controlled between‐subject intranasal OXT administration study. One exclusion criterion was any contraindication for OXT administration. This includes hypersensitivity to intranasally administered OXT (e.g., observed via self‐report or in previous OXT administration studies or the present one by a running and/or snuffling nose after intranasal OXT administration), and nasal congestion. Also neurological or psychiatric disorders (including drug/alcohol abuse), regular or current medication, and participation in another OXT administration study within the last 6 months prior to the present experiment were exclusion criteria. In total, *N* = 176 Chinese males participated in the present experimental study. Of note, the required sample size was estimated from previous studies investigating interaction effects between OXTR genetics and OXT administration (Chen et al., 2015; Feng et al., 2015); however, our sample might still be rather small given the exclusion of some participants detailed further below and the special paradigm, including the specific kind of analysis to evaluate the data, used in the present study (see below and results regarding genetics in the Supplementary Material). Participants were asked to sleep as usual on the day before testing and to abstain from caffeine‐containing beverages on the day of the experiment. Due to technical failures, missing data and/or as a result of misunderstood instructions, *n* = 15 participants (eight receiving placebo [PLC], seven receiving OXT) were excluded from the final analysis leading to a final sample size of *N* = 161 participants (*n* = 80 PLC, *n* = 81 OXT; *M*_age_ = 21.12, *SD* = 2.50; *n* = 149 Han). The study was approved by the local ethics committee at the University of Electronic Science and Technology of China (UESTC), Chengdu, China. The study implementation was in accordance with the latest revision of the Declaration of Helsinki. All participants gave written informed consents prior to participation in both the CGBBP and the present experimental study.

### 2.2 Experimental procedure

#### 2.2.1 Oxytocin challenge study

For participation in the randomized double‐blind placebo‐controlled between‐subject design experimental study, each participant received a basic payment plus the money they individually earned in the economic games (participants also took part in other experiments, which are not of interest for the present research endeavor; among others also a dictator and an ultimatum game after the die‐in‐a‐cup paradigm. Only the die‐in‐a‐cup paradigm results are presented here since the other paradigms have different objectives and involve interactions with others). A between‐subject design was chosen to prevent carry‐over effects when repeatedly participating in the same paradigm (e.g., the die‐in‐a‐cup paradigm; Fischbacher & Föllmi‐Heusi, 2013). Each participant sat in a separated (obscured) cubicle. Hence, neither the experimenters nor the participants could see each other during the experiments. After arriving in the laboratory, participants first filled in some basic demographic information as well as baseline measures for other experimental paradigms (Positive and Negative Affect Schedule (Qin, Zheng, & Wang, 2008), Social Interaction Anxiety Scale (Ye, Qian, Liu, & Chen, 2007), State‐Trait Anxiety Inventory (Li & Qian, 1995), Liebowitz Social Anxiety Scale (He & Zhang, 2004), Inclusion of Other Scale (Zhang, Wang, & Yang, 2006), pretest for an attentional bias paradigm). After a standardized explanation by the experimenters participants got used to using the nasal spray by spreading into a towel. Next, participants self‐administered a single dose of 24 international units (IUs) OXT (Syntocinon Spray; Sichuan Meike Pharmacy Co. Ltd; ingredients: oxytocin solution, glycerin, sodium chloride, and purified water) or PLC intranasally under supervision of the (blinded) trained experimenters, hence, while experimenters were in the room and monitored the administration with three puffs per nostril in interchanging order. The self‐administration is the standard procedure in the field (see, e.g., also Aydogan, Jobst et al., 2017; Riem, Van Ijzendoorn, & Bakermans‐Kranenburg, 2019; Schwaiger, Heinrichs, & Kumsta, 2019; Shalvi & De Dreu, 2014). The supervised self‐administration was employed to increase compliance of the participants with the intranasal administration protocols. Moreover, the sprays were weighted before and after the self‐administration to make sure each participant received a similar amount of PLC/OXT (at least 0.6 g difference between measurements [before and after six puffs]). The PLC‐spray contained all the same ingredients as the OXT‐spray except the neuropeptide (OXT) and was provided by the same company and in the same dispensers as OXT. After self‐application, the participants had to wait for 45 min to start the experiments under the influence of the treatment (PLC or OXT). The complete procedure was in accordance with latest standardization guidelines (Guastella et al., 2013; Kendrick et al., 2017). After waiting 45 min and before the die‐in‐a‐cup paradigm, participants again filled in some questionnaires (Positive and Negative Affect Schedule (Qin et al., 2008), Social Interaction Anxiety Scale (Ye et al., 2007), State‐Trait Anxiety Inventory (Li & Qian, 1995), Liebowitz Social Anxiety Scale (He & Zhang, 2004), Inclusion of Other Scale (Zhang et al., 2006), pretest for an attentional bias paradigm). The die‐in‐a‐cup paradigm itself was implemented around 50–60 min after self‐application and took around 5 min (including instructions; the three rounds of the paradigm to determine the extra payoff [see below] were implemented in around a minute). Current research suggests that OXT effects might be most pronounced in the time of 45–70 min after intranasal administration of 24 IUs (as measured by means of amygdala responsiveness to fear; Spengler et al., 2017). Therefore, the paradigm took part in the potentially most effective time window for this method. The participants were not able to guess better than chance if they received PLC or OXT (Chi^2^(1) = 2.30, *p *= .129 [*N* = 161]), confirming successful double‐blinding.

#### 2.2.2 The die‐in‐a‐cup paradigm

The die‐in‐a‐cup paradigm (similar to the procedure used in, e.g., Fischbacher & Föllmi‐Heusi, 2013; Gächter & Schulz, 2016; Wibral et al., 2012) was explained by standardized on‐screen presentations (in case of problems or questions, the experimenters were available). The participants were first informed that they would receive an additional payoff for the following task according to the numbers they threw on the die (see below). Following this, they were asked to convince themselves that the six‐sided die (which was placed under a black, obscure cup) was not biased in some way by throwing it several times. They were also told explicitly that nobody except themselves could see which numbers they threw; thus, they would have to input the numbers into the computer. To make sure the participants were convinced that nobody could know which numbers they actually threw, it was also explained to them that each participant was instructed to put the die back under the black cup showing the 1 on the upright position after completing the experiment. Next, the payment rules were explained to them: If they threw a 1, they would get 1 monetary unit (MU) extra payoff for entering a 1 in the computer, for a 2, they would get 2 MUs, and so on. Each MU was worth 1 RMB. But if they threw a 6, or rather entered a 6 in the computer, they would get nothing for that round. The participants' decision had no direct influence on other individuals, only on their own payoff. The participants were asked to throw the die three times to determine the extra payoff and place the die with the 1 on the upright position back under the black cup afterwards. While throwing the die and inputting the numbers in the computer, the rule of payment was always displayed on the screen in form of a table. As nobody other than the participants knew, which numbers they actually threw, they could lie regarding the numbers they threw to maximize their payoff. For this paradigm, clearly a larger sample size is necessary as compared to paradigms in which lying is detectable on individual level. Nevertheless, in economics deception is extremely uncommon (see Cooper (2014) for a short introduction/overview). In the present case, we chose the die‐in‐a‐cup paradigm to make sure participants could trust the experimenters, because they were honest when stating that nobody would know which number participants actually diced. Unreliable results through a bias in the participants' behavior due to the fact that they could not trust the experimenters should be avoided through this procedure. After the die‐in‐a‐cup paradigm, participants were asked to rate how honest they thought other participants would be in the die‐in‐a‐cup paradigm on a 7‐point Likert scale (1 = totally dishonest; 7 = totally honest). This was done to assess whether differences in the belief about the honesty of others would be associated with OXT treatment and thereon differences in honesty or lying behavior (on group level; see, e.g., Aydogan, Jobst, et al., 2017; Shalvi & De Dreu, 2014).

### 2.3 Statistical analyses

#### 2.3.1 Analyzing possible confounding/influential variables

Differences between PLC and OXT groups were assessed for age and the Honesty‐Humility (sub)scale(s) of the HEXACO‐PI‐R (Lee & Ashton, 2018). The HEXACO‐PI‐R personality trait questionnaire was assessed during the CGBBP and was therefore not influenced by treatment. Additionally, participant's ratings of honesty of others playing the die‐in‐a‐cup paradigm were compared between the PLC and OXT groups (as a potential confounding variable in the association between treatment and lying behavior). For these analyses  Mann–Whitney *U* tests were used. The reliability of the HEXACO‐PI‐R Honesty‐Humility scale was Cronbach's alpha = 0.74 (Cronbach's alphas for the subscales: Sincerity: 0.57, Fairness: 0.68, Greed‐Avoidance: 0.57, Modesty: 0.51). Nonparametric analyses were chosen because several of the dependent variables did not fulfill criteria for parametric testing. The reported *p*‐values are two‐tailed.

#### 2.3.2 Analyzing the die‐in‐a‐cup paradigm

Analyzing the data of the die‐in‐a‐cup paradigm is only possible on group level as individual lies are undetectable with the present experimental setup. Therefore, the distribution of the reported numbers (numbers inserted in the computer; all three rounds collapsed) was compared with the equal distribution within the treatment groups (PLC vs. OXT). By chance (and after many rounds), each number should have been thrown in 1/6th = 16.67% of the rounds. Moreover, the distributions found in the PLC and OXT groups as well as the actual average claims (the average payoff claimed for one time throwing the die; lies between 0 and 5) were also compared directly between the two groups. Additionally, also the effects of treatment (PLC vs. OXT) on the distributions of reported numbers in each round separately were investigated and compared against the equal distribution. Moreover, the distributions found in each round were directly compared between the PLC and OXT groups. The actual average claim was also compared between the two groups for each round separately.

To test statistically for significance of the deviations from the expected equal distribution, chi‐square tests were calculated. If chi‐square tests revealed significant (*p* < .05, two‐tailed) deviations, the observed frequencies of each individual number were compared with the expected frequency (1/6th) using binomial tests (two‐tailed; see, e.g., Wibral et al. (2012) for a similar approach). To compare the difference of the distributions between the PLC and OXT group, also chi‐square tests were used. To test for significant differences in the actual average claim between the groups, Mann–Whitney *U* tests were used. All reported *p*‐values are two‐tailed.

## 3 RESULTS

### 3.1 Possible confounding/influential variables

Mann–Whitney *U* tests revealed no significant differences between PLC and OXT groups in the possible confounding/influential variables (age: *Z* = −0.62, *p *= .532; HEXACO‐PI‐R Honesty‐Humility: *Z* = −0.78, *p *= .438, Sincerity: *Z* = −1.62, *p *= .106, Fairness: *Z* = −0.43, *p *= .667, Greed‐Avoidance: *Z* = −0.13, *p *= .897, Modesty: *Z* = −0.78, *p *= .439; ratings of how honest other participants were thought to be in the die‐in‐a‐cup paradigm: *Z* = −0.80, *p *= .426). Mean values and standard deviations of these variables are presented in Table 1. As a result of the nonsignificant differences, it was decided not to include these variables as additional variables in further analyses.

**Table 1:** Descriptive statistics of the possible confounding/influential variables of interest split by treatment group

	PLC *M* (*SD*)	OXT *M* (*SD*)
Age	21.25 (2.54)	21.00 (2.47)
Honesty‐Humility	3.06 (0.44)	3.11 (0.43)
Honesty‐Humility Sincerity	2.88 (0.59)	3.00 (0.59)
Honesty‐Humility Fairness	3.27 (0.74)	3.32 (0.74)
Honesty‐Humility Greed‐Avoidance	2.89 (0.64)	2.86 (0.65)
Honesty‐Humility Modesty	3.21 (0.53)	3.27 (0.64)
Rating of honesty of others in the die‐in‐a‐cup paradigm	4.15 (0.73)	4.21 (0.86)

Note

Only the variable on the rating of the honesty of others in the die‐in‐a‐cup paradigm was assessed under the influence of PLC/OXT.

Abbreviations: OXT, oxytocin; PLC, placebo.

### 3.2 Effects of treatment on lying behavior

**Figure 1:**
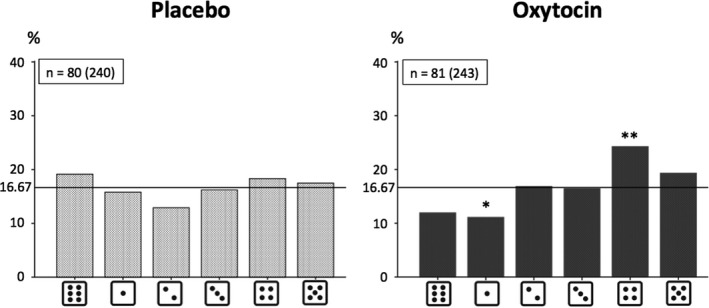
Distributions of numbers reported across the three rounds (in %) in the placebo (PLC) and oxytocin (OXT) groups. Binomial tests were only calculated for the OXT group, where the chi‐square test revealed a significant deviation from the equal distribution: *p < .05, **p < .01, ***p < .001 (two‐tailed); *n* = number of participants in the respective group (number of times the die was thrown = number of participants in the respective group × 3)

When investigating our first hypothesis concerning the effects of treatment on lying behavior, we found that the distribution of the reported numbers (all three rounds collapsed) did not deviate significantly from the equal distribution in the PLC group (Chi^2^(5) = 3.55, *p* = .616). However, in the OXT group there was a significant deviation from the equal distribution (Chi^2^(5) = 17.27, *p* = .004). As can be seen in Figure 1, the observed distribution indicates evidence for lying behavior only in the OXT group. This effect would hold after correction for multiple testing (0.05/2 = 0.025; divided by two because two groups [PLC vs. OXT] were investigated). The difference in the distributions between the PLC and OXT groups failed to be significant (Chi^2^(5) = 9.56, *p* = .089). The difference in the actual average claim between the PLC (*M* = 2.51 MU [*SD* = 1.77]) and OXT (*M* = 2.88 MU [*SD* = 1.64]) groups was significant (*Z* = −2.25, *p* = .024).

**Figure 2:**
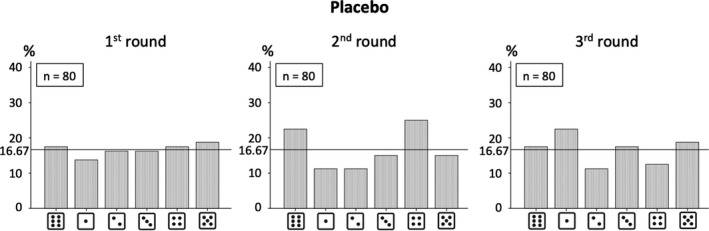
Distributions of numbers reported in the 1st, 2nd, and 3rd round of the die‐in‐a‐cup paradigm (in %) in the placebo (PLC) group. Chi‐square tests revealed no significant deviation from the equal distribution. *n* = number of participants in the respective group

**Figure 3:**
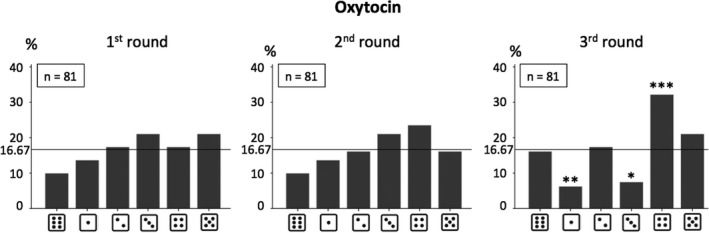
Distributions of numbers reported in the 1st, 2nd, and 3rd round of the die‐in‐a‐cup paradigm (in %) in the oxytocin (OXT) group. Binomial tests were only calculated for the third round as only in this round the chi‐square test revealed a significant deviation from the equal distribution: *p < .05, **p < .01, ***p < .001 (two‐tailed). *n* = number of participants in the respective group

By further investigating each round separately, no significant deviation from the equal distribution in the numbers reported in any round was found in the PLC group (all *p*‐values > .153; see Figure 2). On the other hand and as presented in Figure 3, it was found that lying in the OXT group seemed to be enhanced with each round (in particular with regard to the choice of number 4). The only significant deviation from the equal distribution observed in the OXT group was in the third round (Chi^2^(5) = 22.04, *p* < .001; all other *p*‐values > .317). This effect would also hold after correction for multiple testing (0.05/(2 × 3) = 0.05/6 = 0.0083; divided by 2 × 3 because the effects in two groups [PLC vs. OXT] and three rounds were investigated). In line with this, the only significant difference in the distributions of reported numbers between PLC and OXT groups was found in the third round (Chi^2^(5) = 18.90, *p* = .002). However, the actual average claim did just fail to be significantly different between the groups in the third round (PLC: *M* = 2.41 [*SD* = 1.78], OXT: *M* = 2.96 [*SD* = 1.75]; *Z* = −1.92, *p* = .055). This is most likely due to the lower power compared to the analysis for all rounds collapsed. It should be notet that descriptively the difference between the PLC and OXT groups in the actual average claim of the third round is higher compared to the difference across all rounds collapsed.

## 4 DISCUSSION

The present study sought to investigate potential effects of intranasal OXT administration (and OXTR genetics) on self‐serving lying. A significant effect of intranasal OXT treatment on self‐serving lying behavior (in the absence of a social justification) was observed: For the OXT group, lying could be inferred, whereas this was not true for the PLC group across the three rounds. Whereas the direct comparison of the distributions of reported numbers found in the  PLC versus OXT groups only revealed a nearly significant result, the actual average claim also differed significantly between the two treatment groups across rounds. In the OXT group, lying behavior was particularly increased in the third and final round of the die‐in‐a‐cup paradigm. Specifically, in this round also the distribution of reported numbers differed significantly between the PLC and the OXT group. The actual average claim of the third round just failed to be significantly different between the groups. This also raises potential methodological issues for future studies in which OXT effects on self‐serving behavior or other antisocial behaviors are investigated since these may occur especially pronounced when subjects are given repeated opportunities to display such behaviors.

We argue that the present results can be explained by the social salience hypothesis of OXT and its anxiolytic effects: First, the social cue in the die‐in‐a‐cup paradigm is the potential of being caught lying by the experimenters and being punished, consecutively. Before the first round, this might cause anxiety and participants under the influence of OXT (and PLC) act honestly. However, as the experimenters did not detect or punish lying behavior in the present study, the threatening social cue of potentially being caught lying turned out to be a safety cue with increasing number of rounds. Therefore, participants under the influence of OXT potentially focusing on this social stimulus might have learned that the situation is not dangerous but safe. This in turn might have decreased the fear and ultimately led to increasing self‐serving lying behavior with ascending number of rounds. In contrast, the lack of OXT‐induced anxiolytic effects following PLC may have promoted a continuing social threat across the rounds of the paradigm and honest responses of the participants under the influence of PLC (Shamay‐Tsoory & Abu‐Akel, 2016).

However, previous studies have reported that OXT administration only led to increased lying behavior (compared to PLC) when it benefitted an in‐group (including oneself) and in competitive environments (vs. noncompetitive environments), in which participants were concerned that another person would take the money by lying if they did not also do so. But no effects on pure self‐serving lying behavior in the absence of social justification possibilities were reported (Aydogan, Jobst, et al., 2017; Shalvi & De Dreu, 2014). This might at first glance seem contradictory to the present results. However, to reconcile the studies and results, it is important to note that the decision about whether to lie can be understood as a consequence of a cost–benefit analysis (Mazar et al., 2008). On the negative side (against lying), there are the potential costs of getting caught, which were minimal in the present paradigm since detection of lies was not possible. Also, cognitive dissonance and the need to actualize one's own self‐concept after lying (because dishonesty does not match the self‐concept of oneself as an honest person) are on the negative side (Mazar et al., 2008). On the positive side (pro lying), there is the enhancement of the additional payoffs received. Taking this into account, it becomes clear that in each study, OXT enhanced self‐serving lying only in situations, in which participants could lie to enhance their payoff but still maintain a positive self‐concept: either by the additional payoff for the in‐group or in line with the expected deceptiveness of the opposite player (Aydogan, Jobst, et al., 2017; Shalvi & De Dreu, 2014) or by not lying to the maximum. The latter justification potentially explains the self‐serving lying effects of OXT found in the present study. As can be seen in the distributions of reported numbers in the OXT group (in which lying could be inferred) in the present study, the 4 rather than the 5 was reported most often. The strategy of lying just a “little bit” can be interpreted as a strategy to maintain a positive self‐concept of oneself as an honest person, despite actually lying (Mazar et al., 2008). Of note, such a strategy was not possible in the other OXT administration studies investigating lying behavior as coin‐toss (prediction) tasks were used. Additionally, the findings of the present study are partly in line with results from a cross‐cultural study, in which similar patterns (lying in the die‐in‐a‐cup paradigm with regard to the 4 but not 5) in samples from Ho Chi Minh City, Vilnius, Granada, and Bogota were found, although in a sample from Shanghai the 5 was reported most often (Gächter & Schulz, 2016). Notably, in the present study the effects of OXT on increased lying behavior were not associated with a higher belief that other participants would be dishonest when performing the task (because OXT did not influence the belief). A conformity effect of OXT (as found in Aydogan, Jobst, et al. (2017) and Stallen, De Dreu, Shalvi, Smidts, and Sanfey (2012)) therefore cannot explain the present results; however, the present paradigm did also not include a competitive setting.

Moreover, in the two previous studies in which effects of OXT administration on pure self‐serving lying behavior (in the absence of a social justification) were not detected, results are based on an overall response across several rounds. Hence, significant effects of OXT administration on lying behavior in later rounds might have gone undetected due to nonsignificant effects in earlier rounds. In the present study, the importance of possible alterations in patterns of lying behavior as a function of repeated opportunities to lie was confirmed: The only distribution of the reported numbers, which significantly deviated from the equal distribution, was the one in the third round in the OXT group (although a trend was already visible in the second round). Revisiting Figure 3, it becomes obvious on a descriptive level that the number of 4 inserted into the computer constantly rises over the three rounds. As mentioned above, a possible explanation is that with increasing number of rounds without (negative) consequences of the behavior, certainty that lying would not be detected might be increased and fear of being punished might be reduced under the influence of OXT. Therefore, participants under OXT might tend to lie more. However, with the present study design we cannot test this directly and other explanations are also possible. We did not, for example, obtain self‐reports on how certain a person was that lying would go undetected across the three rounds, because doing so might have altered behavior during the task. It is also noteworthy that in the third round in the OXT group the increased input of the 4 was at the expense of the 1 and the 3, whereas the 2 was reported nearly exactly as often as would be expected by chance. This is a result we cannot explain with the present data. However, it is not uncommon that the report of numbers leading to no or lower payoffs does not show an ascending distribution with increasing payoff in this paradigm (e.g., Gächter & Schulz, 2016).

Several limitations of the present study should also be acknowledged. First, by using the die‐in‐a‐cup paradigm it cannot be examined whether OXT treatment (in interaction with OXTR individual haplotypes) influences the general tendency to maximize one's own profits or to lie. Additionally, comparing distributions is the common way to analyze results of the die‐in‐a‐cup paradigm. However, by doing so it is not possible to model interaction effects between treatment and round. In the present case, it was also not possible to search for interaction effects on payoffs (i.e., the actual average claim) by using an ANOVA approach, because the dependent variable did not fulfill criteria for parametric testing. Additionally, it needs to be mentioned that the equal distribution, to which—among others—the distributions found in the present study were compared, is a theoretically expected frequency when a die is thrown an infinite number of times and participants report the numbers honestly. However, in the present study the die was not thrown an infinite number of times, which is why also the direct comparisons between the distributions found in the PLC and OXT groups are reported. Moreover, one might ask whether participants lied for a pure self‐serving reason or to gain money for their in‐group, family, or children. However, even if possible, we are of the opinion that this is unlikely as no hint toward any social group was given in the experiment. Additionally, most participants were rather young and therefore most likely did not have children. Next, only males were investigated. Since sex‐dependent effects of OXT have been reported by previous studies (Gao et al., 2016; Scheele et al., 2014), it is possible that OXT effects on self‐serving lying might be different in females. However, no females were included due to possible interferences with their hormonal status depending on the individual phase of the menstrual cycle or intake of hormonal contraceptives (see, e.g., fluctuations in OXT plasma levels depending on menstrual cycle and contraceptives (Salonia et al., 2005) or other OXT treatment studies on females discussing this problem (Scheele et al., 2014; Theodoridou, Rowe, Penton‐Voak, & Rogers, 2009)). Moreover, effect sizes might be even higher, if more rounds of the die‐in‐a‐cup paradigm are implemented. Finally, overall the present sample seemed to be quite honest (especially in the PLC group). This might be due to the sample of Chinese students and begs the question on generalizability of the present findings. It is also possible that the participants were not sure about whether lying would *really* not be detected/punished in the beginning. An effect that seems to have been reduced by OXT (but not PLC) especially in association with the rising number of successful rounds of lying. However, as we did not assess whether participants trusted the experimenters or feared being detected lying during each round, we cannot provide further insights. The high degree of honesty might also be due to the special setup of the present study. Other studies investigating the die‐in‐a‐cup paradigm often ask the participants to throw the die several times but only report the first outcome to determine the payoff; the additional die rolls are thought to ensure that the die is not manipulated. Investigating differences in the experimental setups of the die‐in‐a‐cup paradigm, one study found that participants tend to report the highest of all outcomes, even if only the first throw should “count”, while honesty was increased when the die could only be thrown once (Shalvi, Dana, Handgraaf, & De Dreu, 2011); and in the present study, participants could only throw the die one time each round. Moreover, as the experimenters indeed told the truth about not being able to detect lying, they were absolutely trustworthy. This might also be an important example of why a “no deception” rule is of tremendous importance. Only if participants can rely on the honesty of the experimenters, they act unbiased and—as in the present study—gain confidence. On the other hand, if participants cannot trust the experimenters (e.g., because they are indeed not honest), this might lead to effects which were fully unintended. Therefore, it is unavoidable to investigate such paradigms in the absence of any deception if possible.

Lastly, also the recent discussion on intranasally applied OXT and its effectiveness should be discussed briefly. Results on the effects of exogenous OXT administration on psychological functions, social cognition, and behavior in humans seem heterogeneous with several studies showing pro‐ but also several studies showing rather antisocial and immoral effects (Declerck, Boone, & Kiyonari, 2013; Israel, Weisel, Ebstein, & Bornstein, 2012; Kosfeld, Heinrichs, Zak, Fischbacher, & Fehr, 2005; Radke & de Bruijn, 2012; Scheele et al., 2014; Shamay‐Tsoory et al., 2009; Zak, Stanton, & Ahmadi, 2007). These results not only led to the conclusion that the characterization of OXT as “cuddle hormone” might not be completely true, but also that several moderating factors exist, which explain the various effects of OXT administration (Bartz et al., 2011; Shamay‐Tsoory & Abu‐Akel, 2016). As such, person variables (in the present work: Genetics [see Supplementary Material] as well as situational variables (in the present work: repeated opportunities to lie undetectably) are discussed. It also needs to be mentioned that several OXT administration studies suffer from drawbacks such as underpowered sample sizes. Also, the possibility of publication bias, as well as the questions about whether, and how much OXT enters the brain represent important topics in the field of OXT research (Leng & Ludwig, 2016; Walum, Waldman, & Young, 2016). Nevertheless, there is strong indication that intranasally administered OXT enters the brain (at least in parts) and has effects on brain regions involved in social cognition and emotion (Paloyelis et al., 2016; Valstad et al., 2017).

## 5 CONCLUSIONS

In conclusion, the findings of the present study demonstrate that OXT plays an important role in self‐serving lying and thereon potentially also other (anti‐)social behaviors. Moreover, these effects of intranasal OXT administration are highly specific and situational factors, such as the repeated chance to lie undetected, seem to influence them. In the Supplementary Material, also potential moderating effects of genetic underpinnings of the OXTR gene are discussed. Therefore, to gain a deeper understanding of the effects of OXT treatment, and to help interpret heterogeneous results in the literature, it may help if future OXT administration studies also take into account more situational factors and/or the genotypes of participants.

## CONFLICT OF INTEREST

None.

## AUTHORS' CONTRIBUTIONS

CS and CM planned the design of the present study. BB and KMK gave helpful advice to improve the study design. CS and RL programmed the paradigms and assessed the data (genetic as well as experimental). CS and RL conducted the genetic analyses. CS wrote the manuscript and carried out the statistical analyses. CM, BB, and KMK worked over the manuscript. All authors read and approved the final manuscript.

## Data Availability

The datasets generated and/or analyzed during the current study are not publicly available due to the fact that the data are extremely sensitive (e.g., information on several genetic markers) and participants did not explicitly give their consent for publication of their data. The datasets used and/or analyzed during the current study are available from the corresponding author on reasonable request.
