# Supplementary material for: The role of oxytocin on self‐serving lying
Source: Brain Behav. 2020 Jan 13;10(2):e01518. doi: 10.1002/brb3.1518 (PMC7010580; doi:10.1002/brb3.1518)
Supplement: Supplementary file 1 [file BRB3-10-e01518-s001.pdf]

The role of oxytocin on self-serving lying

Supplementary Material

Cornelia Sindermann[a]*, Ruixue Luo[b], Benjamin Becker[b], Keith M. Kendrick[b] & Christian Montag[a,b]

[a] Department of Molecular Psychology, Institute of Psychology and Education, Ulm University, 89081 Ulm, Germany

[b] The Clinical Hospital of Chengdu Brain Science Institute, MOE Key Laboratory for Neuroinformation, University of Electronic Science and Technology of China, Chengdu, China

*** Correspondence:**

Cornelia Sindermann

Department of Molecular Psychology, Institute of Psychology and Education, Ulm University, Zentrum für Biomedizinische Forschung, Helmholtzstr. 8/1, 89081 Ulm, Germany.

Phone: +49 (0)731 / 50 26558; E-Mail: cornelia.sindermann@uni-ulm.de

Supplementary Material

Genetics – Background, Methods, Results, and Discussion:

Background:

Heritability estimates of lying are around 29-42% (Eaves et al., 1999). Hence, variations in the oxytocin receptor (OXTR) gene may influence the propensity for self-serving lying. And the impact of intranasal oxytocin (OXT) administration on self-serving lying might be moderated by genetic underpinnings of the OXTR. Support comes from studies reporting moderating effects of polymorphisms in the OXTR gene, located at chromosome 3p25.3 (The National Center for Biotechnology Information, n.D.), on OXT administration effects on various measures of social behavior and cognition (Chen et al., 2015; Feng et al., 2015; Montag, Sauer, Reuter, & Kirsch, 2013). But to date no study has investigated such interaction effects on self-serving lying. Therefore, whereas the hypothesis *that* OXTR genetics moderate the effects of OXT administration seems straight forward, the question about which exact polymorphisms influence the OXT administration effects and in which way has to be investigated exploratively.

Methods:

Genotyping and haplotype analyses

DNA was extracted and purified from buccal cells using a MagNa Pure 96 robot (Roche Diagnostics, Mannheim, Germany; RRID:SCR_001326). Polymorphisms were analyzed by means of polymerase chain reaction and subsequent high-resolution melting on a Cobas LightCycler z480 (Roche Diagnostics, Mannheim, Germany; RRID:SCR_001326). Simple probe assay designs provided by TibMolBiol (Berlin, Germany) were used. The following SNPs were analyzed for each participant included in this study: OXTR rs237887, rs2268491, rs2254298, rs53576, rs2268498.

Linkage disequilibria (LDs) between SNPs in the OXTR gene were identified in the N = 161 participants using Haploview software (Barrett, Fry, Maller, & Daly, 2004; RRID:SCR_003076). Haplotype blocks were built by means of the solid spine of LD method. Individual haplotypes (in the N = 161 participants) were calculated using PHASE v 2.1 software (Stephens, Smith, & Donnelly, 2001; Stephens & Scheet, 2005), which reconstructs haplotypes from population data.

Supplementary Material

Distribution of genotypes, HWE, and haplotypes

As seen in Supplementary Table 1, all distributions of genotypes were in the Hardy-Weinberg-Equilibrium (HWE) for all SNPs.

Supplementary Table 1

Distribution of genotypes in the sample of N = 161 participants

	Genotypes				HWE
rs237887	AA: 32	AG: 83	GG: 46	Total: 161	$Chi^2 = 0.24$, p = .621
rs2268491	CC: 83	CT: 63	TT: 14	Tot0l: 160	$Chi^2 = 0.17$, p = .680
rs2254298	AA: 15	AG: 63	GG: 83	Total: 161	$Chi^2 = 0.36$, p = .547
rs53576	AA: 87	AG: 58	GG: 11	Total: 156	$Chi^2 = 0.10$, p = .755
rs2268498	CC: 18	CT: 70	TT: 72	Total: 160	$Chi^2 = 0.03$, p = .874

Note. The differences in the total N are due to not detected genotypes in some SNPs for some participants; alleles are derived from 5'-3' strand.

Two different haplotype blocks could be identified comprising i) rs237887, rs2268491, rs2254298 and ii) rs53576, rs2268498 (please see Supplementary Figure 1 for LDs).

Regarding the rs237887-rs2268491-rs2254298 haplotype block, five individual haplotypes were detected with only three individual haplotypes of interest due to the number of people carrying the respective individual haplotypes: the GCG-, GTA- and the ACG- individual haplotypes. Within the rs53576-rs2268498 haplotype block, four individual haplotypes could be detected with also three individual haplotypes of interest: the AC-, AT- and GT- individual haplotypes. Compared to the GCG-, GTA-, ACG- (rs237887-rs2268491-rs2254298), AC-, AT-, and GT- (rs53576-rs2268498) individual haplotypes the other individual haplotypes were only rarely found (<5%). Therefore, statistical testing would not be meaningful, which is why they were not considered in further analyses. Haplotype frequencies are presented in Supplementary Table 2. For each of the individual haplotypes groups of

carriers and non-carriers were built. Supplementary Table 3 shows the number of participants in each (sub-)sample analyzed with regard to the behavior in the die-in-a-cup paradigm.

Of note, the genotypes of each individual SNP are not equally distributed for most of the SNPs (see Supplementary Table 1). Accordingly, when splitting the sample into genotype and treatment groups some of the resulting subsample sizes are extremely small (e. g. 6 participants carrying the AA genotype in the rs2254298 and receiving OXT treatment). Therefore, it was decided to not report results of interaction effects between treatment (PLC vs. OXT) and genotype of each single SNP. Instead, it was decided to focus on the results regarding the individual (OXTR) haplotypes.

Of final note: The experiment was carried out blinded for the genetic data.

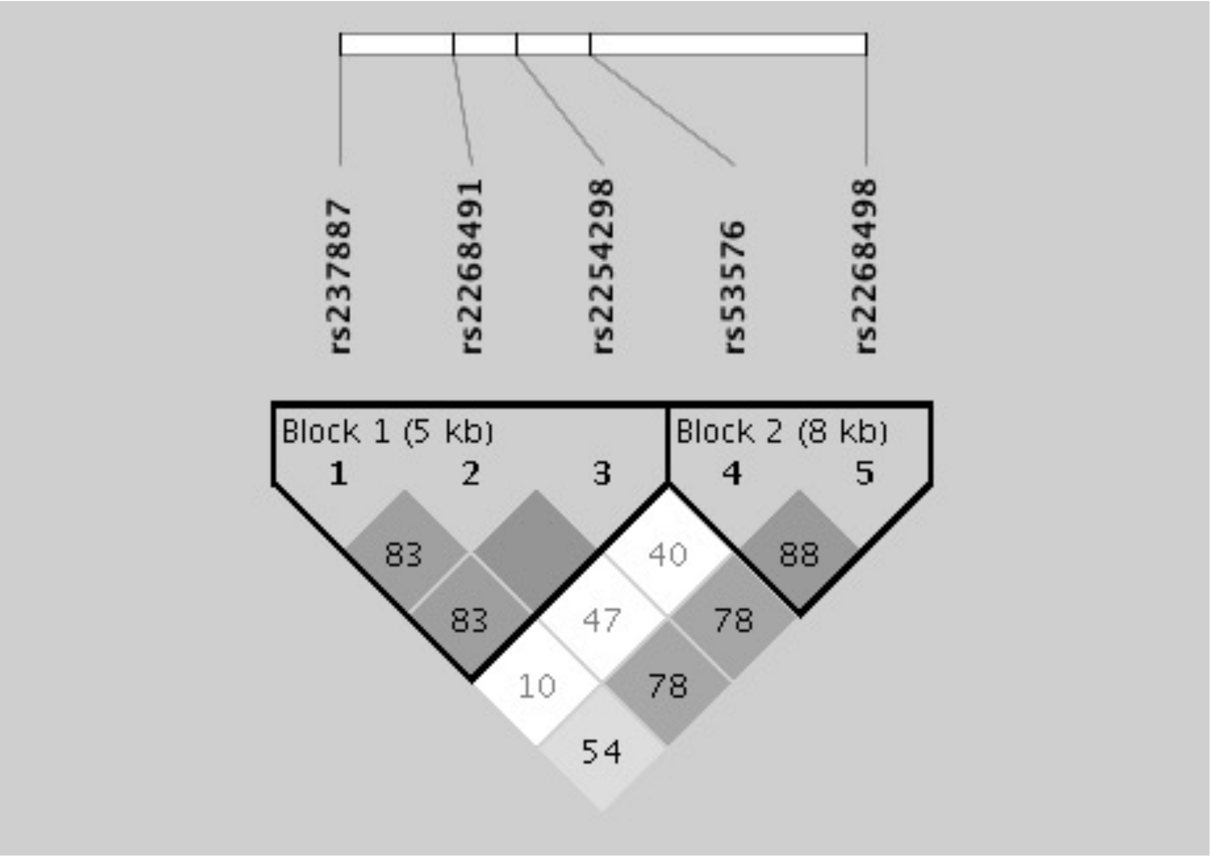

Supplementary Figure 1. Linkage disequilibria between the five OXTR SNPs under investigation and haplotype blocks (N = 161).

Supplementary Material

Supplementary Table 2

Frequencies of individual haplotypes in the sample of N = 161 participants

rs237887-rs2268491-rs2254298		rs53576-rs2268498	
individual haplotype	observed n (%)	individual haplotype	observed n (%)
GCG	87 (27.02)	AC	105 (32.61)
GTA	87 (27.02)	AT	136 (42.24)
ACG	142 (44.10)	GT	79 (24.53)
ATA	5 (1.55)	GC	2 (0.62)
GCA	1 (0.31)		

Note. Numbers sum up to 322 because 161 participants carrying two chromosomes each (2 × 161 = 322) were investigated. Possible individual haplotypes, which are not presented, have not been detected in the present sample.

Supplementary Table 3

Different (sub-)sample sizes analyzed in the die-in-a-cup paradigm

	PLC		OXT	
	carriers	non-carriers	carriers	non-carriers
	rs237887 - rs2268491 - rs2254298			
GCG	36	44	41	40
GTA	32	48	43	38
ACG	59	21	54	27
	rs53576 -rs2268498			
AC	45	35	42	39
AT	52	28	60	21
GT	35	45	35	46

Note. Even if single genotypes in single SNPs could not be determined for some individuals, the statistical procedure to calculate individual haplotypes is still working and providing individual haplotypes including values for all SNPs of the respective haplotype block.

Supplementary Material

Statistical Analyses

Confounding variables

First, the groups of carriers versus non-carriers of all six individual haplotypes were compared regarding age and the Honesty-Humility (sub)scales of the HEXACO-PI-R (Lee & Ashton, 2016). The HEXACO-PI-R personality trait questionnaire was assessed during the CGBBP and was therefore not influenced by treatment; hence, examining genetic effects across treatment groups is justified. These analyses were implemented using Mann-Whitney U tests.

The groups split by PLC and OXT treatment were compared as explained in the main manuscript.

Finally, the groups split by PLC versus OXT treatment and individual haplotype carriers versus non-carriers (2×2 designs) were compared in light of all the previously mentioned possible confounding variables (always comparing 4 groups: PLC, non-carriers vs. PLC, carriers vs. OXT, non-carriers vs. OXT, carriers; for each of the six individual haplotypes) using Kruskal-Wallis tests.

The die-in-a-cup paradigm

Based on the round-specific effects of OXT on lying behavior (see main manuscript), the effects of PLC versus OXT treatment and the six individual haplotypes were analyzed on each round separately. Therefore, the distributions of reported numbers in each separate round split by treatment (PLC vs. OXT) and individual haplotypes (carriers vs. non-carriers; 2×2 designs) were investigated. To test statistically for significance of the deviations from the expected equal distribution, Chi^2-tests were calculated. If Chi^2-tests revealed significant ($p < .05$, two-tailed) deviations, the observed frequencies of each individual number were compared with the expected frequency ($1/6^{th}$) using binomial tests (two-tailed) (see for example Wibral, Dohmen, Klingmüller, Weber, and Falk (2012) for a similar approach). Chi^2-tests were also implemented to compare the distributions found in carriers of the respective individual haplotype in the OXT versus the PLC group and the distributions found in non-carriers of the respective individual haplotype in the OXT versus the PLC group.

Results:

Confounding Variables

No significant differences in age or the HEXACO-PI-R (Lee & Ashton, 2016) Honesty-Humility (sub)scale(s) were observed between the individual haplotype groups (for each of the six individual haplotypes comparing carriers vs. non-carriers), which would hold after correction for multiple testing (0.05 / 6 = 0.0083; divided by six because six individual haplotypes on the OXTR gene were investigated) (all p-values > .035).

No significant differences between OXT and PLC group were observed in the possible confounding variables (see paragraph 3.1 in the main manuscript).

In the groups split by treatment (PLC vs. OXT) as well as carriers and non-carriers of each of the six individual haplotypes (2×2 designs), Kruskal-Wallis tests showed no significant difference in the possible confounding variables, which would hold after correction for multiple testing (0.05/(6×2) = 0.05/12 = 0.0042; divided by 6×2 because six individual haplotypes on the OXTR gene were investigated in two groups each (PLC and OXT) – all p-values > .008). As a result of this, it was decided not to include these variables as confounding variables in further analyses.

Results of effects of treatment and individual haplotype on each round separately

In the PLC group, no meaningful effect of any individual haplotype (investigating carriers and non-carriers) was found on any round (only GT non-carriers under PLC showed a deviation from the equal distribution in the third round with a p = .049; the other respective Chi^2-tests revealed p-values > .083). In the OXT group significant results were observed, particularly in the third round (only one significant effect in the second round (p < .05), but none in the first round). In detail, a significant deviation of the distribution of numbers reported in the third round from the equal distribution could be detected in several groups. No significant differences were observed in the distributions between OXT group and PLC group (comparing the respective carriers (with carriers) and non-carriers (with non-carriers) of the OXT and PLC groups) in the first and second round. However, several significant differences were observed in the third round. These results are presented in Supplementary Table 4.

Supplementary Material

Supplementary Table 4

Statistics for the deviation of the distributions of reported numbers in the OXT group in the third round from the equal distribution and the distributions in the respective PLC group for carriers and non-carriers of the individual haplotypes of interest

	OXT vs. equal distribution		OXT vs. PLC	
	carriers	non-carriers	OXT, carriers (vs. PLC, carriers)	OXT, non-carriers (vs. PLC, non-carriers)
rs237887 - rs2268491 - rs2254298				
GCG	**$Chi^2(5)=20.61$, $p<.001$**	$Chi^2(5)=6.80$, $p=.236$	**$Chi^2(5)=12.55$, $p=.028$**	$Chi^2(5)=10.74$, $p=.057$
GTA	$Chi^2(5)=8.21$, $p=.145$	$Chi^2(5)=19.47$, $p=.002$	$Chi^2(5)=10.67$, $p=.058$	**$Chi^2(5)=13.57$, $p=.019$**
ACG	$Chi^2(5)=19.56$, $p=.002$	$Chi^2(5)=3.89$, $p=.566$	**$Chi^2(5)=16.11$, $p=.007$**	$Chi^2(5)=6.54$, $p=.257$
rs53576 - rs2268498				
AC	$Chi^2(5)=16.00$, $p=.007$	$Chi^2(5)=11.62$, $p=.040$	**$Chi^2(5)=15.93$, $p=.007$**	$Chi^2(5)=7.88$, $p=.163$
AT	$Chi^2(5)=19.40$, $p=.002$	$Chi^2(5)=1.62$, $p=.805$	**$Chi^2(5)=13.52$, $p=.019$**	$Chi^2(5)=9.65$, $p=.086$
GT	$Chi^2(5)=7.00$, $p=.221$	**$Chi^2(5)=21.04$, $p<.001$**	$Chi^2(5)=5.21$, $p=.390$	**$Chi^2(5)=15.66$, $p=.008$**

Note. It is important to keep in mind that when comparing OXT carriers / non-carriers vs. PLC carriers / non-carriers, each OXT sub-group is compared with another reference group (i.e., always the PLC sub-group of participants (not) carrying the respective individual haplotype).

As can be seen in Supplementary Table 4, the strongest effects were found in the groups of GCG (rs237887-rs2268491-rs2254298) carriers and GT (rs53576-rs2268498) non-carriers when comparing the actual distribution versus the expected equal distribution. Only in these groups, the effects would also hold after strict correction for multiple testing ($0.05/(6\times2\times3) = 0.05/36 = 0.0014$; divided by $6\times2\times3$ because the effects of six individual haplotypes in two groups (PLC vs. OXT) on three rounds were investigated). Therefore, these distributions are also shown in Supplementary Figures 2 and 3. When

comparing OXT versus PLC groups, also significant results can be observed. However, when applying strict Bonferroni correction for multiple testing (0.05/(6×3) = 0.05/18 = 0.0028; divided by 6×3 because the effects of six individual haplotypes in three rounds were investigated) none of the effects remains significant.

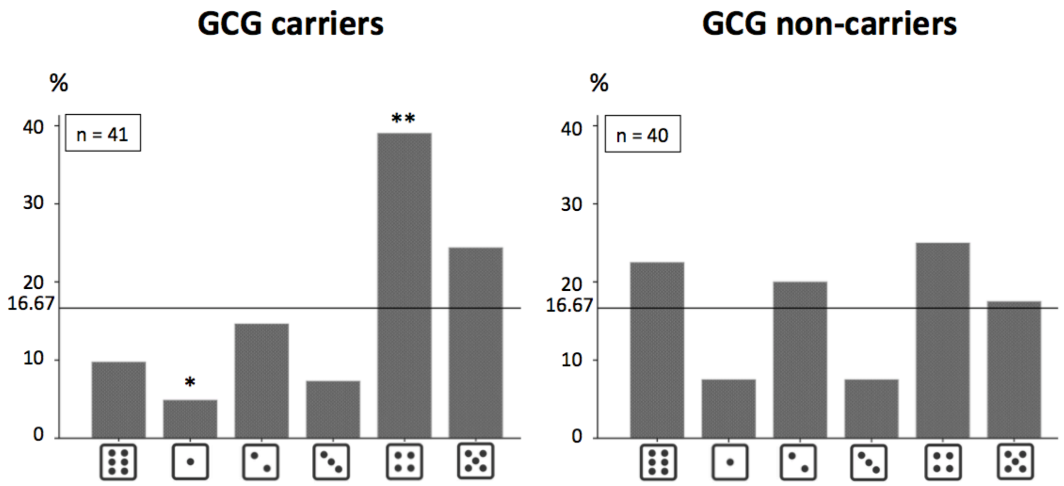

Supplementary Figure 2. Distributions of numbers reported in the 3rd round (in %) in GCG individual haplotype (rs237887-rs2268491-rs2254298) carriers and non-carriers in the OXT group. Binomial tests were only calculated for the carriers group as only in this group the Chi^2-test revealed a significant deviation from the equal distribution: *p < .05, **p < .01, ***p < .001 (two-tailed); n = number of participants in the respective group.

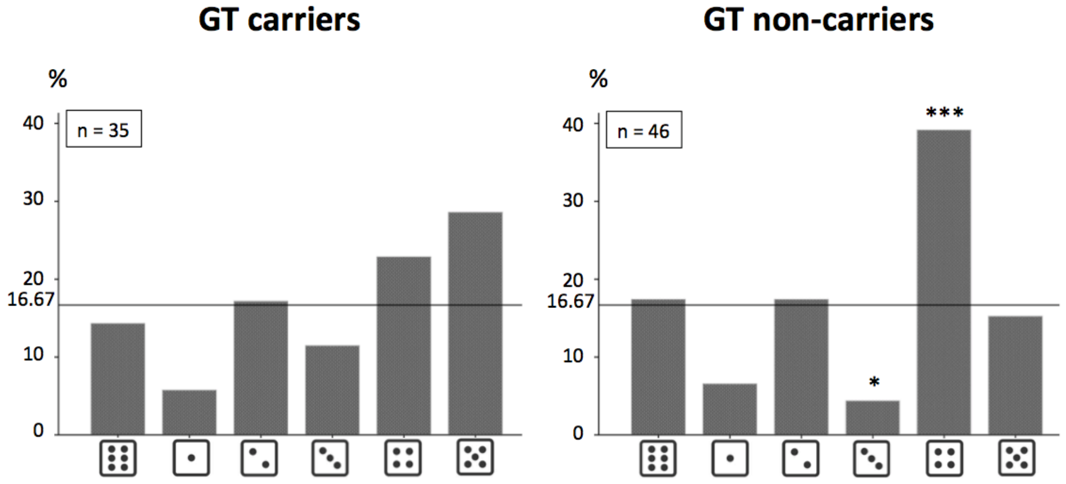

Supplementary Figure 3. Distributions of numbers reported in the 3rd round (in %) in GT individual haplotype (rs53576-rs2268498) carriers and non-carriers in the OXT group. Binomial tests were only calculated for the non-carriers group as only in this group the Chi^2-test revealed a significant deviation from the equal distribution: *p < .05, **p < .01, ***p < .001 (two-tailed); n = number of participants in the respective group.

Additional Post-Hoc Analyses

Next to the GCG (rs237887-rs2268491-rs2254298) carriers and the GT (rs53576-rs2268498) non-carriers, also in the groups of GTA non-carriers and ACG carriers (rs237887-rs2268491-rs2254298) as well as in the groups of AC and AT carriers (rs53576-rs2268498) receiving OXT, highly significant results with regard to the deviation of the distributions of reported numbers in the third round from the equal distribution were found (see Supplementary Table 4). To examine these effects in more detail and to check whether the significant results in these groups would be driven by the participants who also belong to the carriers or non-carriers groups of the individual haplotypes, in which we found the strongest results in the respective block (namely GCG (rs237887-rs2268491-rs2254298) carriers or GT (rs53576-rs2268498) non-carriers), we implemented additional analyses. Therefore, we split each of the groups (receiving OXT + being GTA non-carrier, receiving OXT + being ACG carrier (for the block comprising rs237887-rs2268491-rs2254298); receiving OXT + being AC carrier, receiving OXT +

being AT carrier (for the block comprising rs53576-rs2268498)) additionally into carriers versus non-carriers of the GCG (rs237887-rs2268491-rs2254298) or GT (rs53576-rs2268498) individual haplotype, respectively, and investigated the distributions of reported numbers from the third round. Results of the Chi^2-tests are presented in Supplementary Table 5. By additionally visually investigating the distributions of reported numbers in the third round in these subgroups, it can be concluded that the effects of increased lying behavior found in GTA non-carriers and ACG carriers (rs237887-rs2268491-rs2254298) presented in Supplementary Table 4 are due to the significant results in the subgroup of participants also carrying the GCG (rs237887-rs2268491-rs2254298) individual haplotype. Additionally, the results also underline that the effects of increased lying behavior found in AC and AT (rs53576-rs2268498) carriers presented in Supplementary Table 4 are due to the significant results in the subgroup of participants (also) not carrying the GT (rs53576-rs2268498) individual haplotype. However, it needs to be noted that the final sample sizes for these analyses are small. Therefore, results should be treated cautiously.

Supplementary Table 5

Statistics for the deviation of the distributions of reported numbers in the third round from the equal distribution in the OXT group for the carriers and non-carriers of the individual haplotypes in which significant effects were reported and additionally split by GCG (rs237887-rs2268491-rs2254298) or GT (rs53576-rs2268498) individual haplotype carriers versus non-carriers

	OXT	
rs237887 - rs2268491 - rs2254298	GCG carriers	GCG non-carriers
GTA non-carriers	$Chi^2(5)=22.56$, $p<.001$	$Chi^2(4)=2.18$, $p=.702$
ACG carriers	$Chi^2(5)=17.80$, $p=.003$	$Chi^2(5)=7.65$, $p=.177$
rs53576 - rs2268498	GT carriers	GT non-carriers
AC carriers	$Chi^2(4)=1.56$, $p=.817$	$Chi^2(5)=13.36$, $p=.020$
AT carriers	$Chi^2(5)=3.09$, $p=.686$	$Chi^2(5)=21.05$, $p<.001$

Note. If dfs of a Chi^2-test are 4, the 3 was not reported in the respective group. Group sizes: n(GTA non-carriers + GCG carriers) = 27, n(GTA non-carriers + GCG non-carriers) = 11, n(ACG carriers + GCG carriers) = 20, n(ACG carriers + GCG non-carriers) = 34; n(AC carriers + GT carriers) = 9, n(AC carriers + GT non-carriers) = 33, n(AT carriers + GT carriers) = 22, n(AT carriers + GT non-carriers) = 38.

Supplementary Material

Discussion:

Our results demonstrate potential interactions between OXT treatment and OXTR genetics with the intranasal OXT effect on lying behavior potentially being specific to GCG (rs237887-rs2268491-rs2254298) carriers and GT (rs53576-rs2268498) non-carriers. In detail, OXT treatment seems to enhance lying behavior only in people carrying certain individual haplotypes in the OXTR gene (hence, in the PLC group no effect of individual haplotypes could be observed). The direct comparison of OXT and PLC sub-groups also revealed several significant results. However, none would hold after correction for multiple testing.

Additionally, except rs2268498 (Reuter et al., 2017), the OXTR SNPs under investigation are placed in an intronic region of the gene. Accordingly, their functionality on a biochemical level is unknown. Thus, it is not possible to directly conclude potential functional differences between the groups of certain individual haplotype carriers versus non-carriers. Nevertheless, findings from animal models throw possible light on this issue. For example, a first study in prairie voles indicates that variation in intronic regions of the OXTR, especially in / near so called cis-regulatory elements (regulatory elements in non-coding sequences of the DNA (e. g. intronic regions)), might contribute to differences in OXTR expression especially in brain regions associated with social attachment (King, Walum, Inoue, Eyrich, & Young, 2016). This might be also of importance for explaining the present interaction effects between individual OXTR haplotypes and OXT treatment on lying behavior. Thus, higher transcription and translation of the OXTR gene finally leading to higher expression of OXTRs in brain areas associated with social behaviors, such as (self-serving) lying, might lead to a more efficient processing of the additional externally applied OXT in carriers of certain genotypes / individual haplotypes. However, this cannot be tested with the present data or samples collected and clearly many other mechanisms are possible. Therefore, the mechanisms underlying the individual haplotype by treatment effects found in the present study remain unclear for now. Nevertheless, since no overall differences were observed between the samples (split by treatment and/or individual haplotypes) in stable personality variables (HEXACO-PI-R Honesty-Humility (sub)scales; see paragraphs *2.1 Participants*, *2.3.1 Analyzing possible confounding/influential variables*, and *3.1 Possible confounding/influential variables*), and as lying could not be inferred in any (sub-)group under the influence of PLC but only for specific individual

haplotype groups under the influence of OXT, it is reasonable to conclude that observed effects are indeed caused by an interaction of OXT treatment and OXTR genetics. It needs to be noted though that the actual interaction effect could not be tested given i) the comparison of distributions, which does (as far as we know) not allow for modelling interaction effects and ii) the lack of fulfilled requirements for parametric tests of a comparison of the actual average claims (see also discussion in the main manuscript). Next, rather small sample sizes in some of the subgroups could have influenced our ability to detect effects. But by grouping subjects into carriers versus non-carriers of individual haplotypes this problem was minimized. Hence, it is unlikely that the present results are an artefact of low sample size. Nevertheless, as can also be seen in the distributions, some seem rather "inconsistent". This might be due to the fact, that with such a small number of participants (times the die was thrown) actually no equal distribution can be expected. To validly expect an equal distribution (and test the actual distribution against the equal distribution) potentially more observations as the ones presented / analyzed in the present treatment by genetics interaction design would be necessary. Finally, it is likely that there are additional genes and polymorphisms, which could influence the effects of intranasally applied OXT (e. g. polymorphisms in the CD38 (Cluster of Differentiation 38) gene or in the AVPR1a (arginine-vasopressin receptor 1a) gene (see for example (Israel et al., 2008; Neumann & Landgraf, 2012; Sauer, Montag, Worner, Kirsch, & Reuter, 2012; Stoop, 2012)). For the present study, however, it was decided to investigate only well-established candidate gene polymorphisms of the oxytocinergic system with respect to lying, which have previously been associated with social cognition and relevant behaviors and/or have a known biochemical function (e. g. (Bakermans-Kranenburg & van Ijzendoorn, 2014; Reuter et al., 2017; Walter et al., 2012)).

Overall the present results support the assumption that OXT effects might depend on genetic predispositions of the OXTR gene. This underlines the importance of assessing genetic moderators in OXT administration studies (Bartz, Zaki, Bolger, & Ochsner, 2011; Chen et al., 2015; Feng et al., 2015; Montag et al., 2013; Shamay-Tsoory & Abu-Akel, 2016). Nevertheless, given shortcomings of the present study replication studies are urgently needed.

References

Bakermans-Kranenburg, M. J., & van Ijzendoorn, M. H. (2014). A sociability gene? meta-analysis of oxytocin receptor genotype effects in humans. *Psychiatric Genetics, 24*(2), 45-51. https://doi.org/10.1097/YPG.0b013e3283643684

Barrett, J. C., Fry, B., Maller, J., & Daly, M. J. (2004). Haploview: Analysis and visualization of LD and haplotype maps. *Bioinformatics, 21*(2), 263-265. https://doi.org/10.1093/bioinformatics/bth457

Bartz, J. A., Zaki, J., Bolger, N., & Ochsner, K. N. (2011). Social effects of oxytocin in humans: Context and person matter. *Trends in Cognitive Sciences, 15*(7), 301-309. https://doi.org/10.1016/j.tics.2011.05.002

Chen, F. S., Kumsta, R., Dvorak, F., Domes, G., Yim, O., Ebstein, R. P., & Heinrichs, M. (2015). Genetic modulation of oxytocin sensitivity: A pharmacogenetic approach. *Translational Psychiatry, 5*(10), e664. https://doi.org/10.1038/tp.2015.163

Eaves, L., Heath, A., Martin, N., Maes, H., Neale, M., Kendler, K., . . . Corey, L. (1999). Comparing the biological and cultural inheritance of personality and social attitudes in the Virginia 30 000 study of twins and their relatives. *Twin Research and Human Genetics, 2*(2), 62-80. https://doi.org/10.1375/136905299320565933

Feng, C., Lori, A., Waldman, I. D., Binder, E. B., Haroon, E., & Rilling, J. K. (2015). A common oxytocin receptor gene (OXTR) polymorphism modulates intranasal oxytocin effects on the neural response to social cooperation in humans. *Genes, Brain and Behavior, 14*(7), 516-525. https://doi.org/10.1111/gbb.12234

Israel, S., Lerer, E., Shalev, I., Uzefovsky, F., Reibold, M., Bachner-Melman, R., . . . Yirmiya, N. (2008). Molecular genetic studies of the arginine vasopressin 1a receptor (AVPR1a) and the oxytocin receptor (OXTR) in human behaviour: From autism to altruism with some notes in between. *Progress in Brain Research, 170*, 435-449. https://doi.org/10.1016/S0079-6123(08)00434-2

King, L. B., Walum, H., Inoue, K., Eyrich, N. W., & Young, L. J. (2016). Variation in the oxytocin receptor gene predicts brain region–specific expression and social attachment. *Biological Psychiatry, 80*(2), 160-169. https://doi.org/10.1016/j.biopsych.2015.12.008

Lee, K., & Ashton, M. C. (2016). Psychometric properties of the HEXACO-100. *Assessment,* 1073191116659134. https://doi.org/10.1177/1073191116659134

Montag, C., Sauer, C., Reuter, M., & Kirsch, P. (2013). An interaction between oxytocin and a genetic variation of the oxytocin receptor modulates amygdala activity toward direct gaze: Evidence from a pharmacological imaging genetics study. *European Archives of Psychiatry and Clinical Neuroscience, 263*(2), 169-175. https://doi.org/10.1007/s00406-013-0452-x

Neumann, I. D., & Landgraf, R. (2012). Balance of brain oxytocin and vasopressin: Implications for anxiety, depression, and social behaviors. *Trends in Neurosciences, 35*(11), 649-659. https://doi.org/10.1016/j.tins.2012.08.004

The National Center for Biotechnology Information (n.D.). OXTR oxytocin receptor [homo sapiens (human)]. Retrieved from https://www.ncbi.nlm.nih.gov/gene/5021

Reuter, M., Montag, C., Altmann, S., Bendlow, F., Elger, C., Kirsch, P., . . . Weber, B. (2017). Functional characterization of an oxytocin receptor gene variant (rs2268498) previously associated with social cognition by expression analysis in vitro and in human brain biopsy. *Social Neuroscience, 12*(5), 604-611. https://doi.org/10.1080/17470919.2016.1214174

Sauer, C., Montag, C., Worner, C., Kirsch, P., & Reuter, M. (2012). Effects of a common variant in the CD38 gene on social processing in an oxytocin challenge study: Possible links to autism. *Neuropsychopharmacology : Official Publication of the American College of Neuropsychopharmacology, 37*(6), 1474-1482. https://doi.org/10.1038/npp.2011.333

Shamay-Tsoory, S. G., & Abu-Akel, A. (2016). The social salience hypothesis of oxytocin. *Biological Psychiatry, 79*(3), 194-202. https://doi.org/10.1016/j.biopsych.2015.07.020

Stephens, M., & Scheet, P. (2005). Accounting for decay of linkage disequilibrium in haplotype inference and missing-data imputation. *The American Journal of Human Genetics, 76*(3), 449-462. https://doi.org/10.1086/428594

Stephens, M., Smith, N. J., & Donnelly, P. (2001). A new statistical method for haplotype reconstruction from population data. *The American Journal of Human Genetics, 68*(4), 978-989. https://doi.org/10.1086/428594

Stoop, R. (2012). Neuromodulation by oxytocin and vasopressin. *Neuron, 76*(1), 142-159. https://doi.org/10.1016/j.neuron.2012.09.025

Walter, N. T., Montag, C., Markett, S., Felten, A., Voigt, G., & Reuter, M. (2012). Ignorance is no excuse: Moral judgments are influenced by a genetic variation on the oxytocin receptor gene. *Brain and Cognition, 78*(3), 268-273. https://doi.org/10.1016/j.bandc.2012.01.003

Wibral, M., Dohmen, T., Klingmüller, D., Weber, B., & Falk, A. (2012). Testosterone administration reduces lying in men. *PloS One, 7*(10), e46774. https://doi.org/10.1371/journal.pone.0046774
